# Supplementary material for: Transcriptomic Analysis of Radish (Raphanus sativus L.) Roots with CLE41 Overexpression
Source: Plants (Basel). 2022 Aug 20;11(16):2163. doi: 10.3390/plants11162163 (PMC9416626; doi:10.3390/plants11162163)
Supplement: Supplementary file 1 [file plants-11-02163-s001.zip › Table_S2.pdf]

Table S2. A list of primers designed to verify the results of RNA-seq analysis.

Gene name	NCBI identifier	Amplicon size, bp	Orientation	Primer sequence, 5'-3'	GC,%	Tm,°C
**Target genes**						
Upregulated genes						
RsDC8	XM_018626573.1	188	Forward	CGAGGCAGGAGAAGAAGCA	57.89	61.77
			Reverse	TCCCTAACGCACCCCTTACC	60.00	63.32
RsP8B6	XM_018604462.1	163	Forward	AGGTGGGACTGGAGGCAAA	57.89	62.98
			Reverse	CTCGGCGTCTTCCTTGTCAG	60.00	63.38
RsXERO1	XM_018581961.1	235	Forward	GGTGGTGGTGGTTTGAGTGG	60.00	63.62
			Reverse	TGATGCCTTTCTTCTCGTGGT	47.62	62.09
RsOLE2	XM_018628424.1	211	Forward	TGGCTATGGTGGTGGTTATGG	52.38	62.79
			Reverse	CGGGCTGAAGATCAGGAAAA	50.00	62.53
RsMYC2	XM_018595757.1	215	Forward	CAAAGGAAAGCCCAAACAGAGA	45.45	62.79
			Reverse	TACCCGCCAACCCAGCAC	66.67	65.39
RsCycU2	XM_018633306.1	186	Forward	TTCTGTTCTCGGTGGGGTTC	55.00	62.38
			Reverse	GCTGGTGGTGGTGATGATGTT	52.38	63.16
RsWRKY40	XM_018616745.1	162	Forward	ACGACAACTGCTTTGGTGGA	50.00	61.69
			Reverse	TTTCTTGGGAGGACTGACTTGG	50.00	62.75
Downregulated genes						
RsPIR2	XM_018578727.1	176	Forward	GCTGGTGATGTTCAGTGGATG	52.38	61.54
			Reverse	CCGTTTTCTTCTGCTTTTGGT	42.86	60.63
RsSRC2	XM_018592015.1	156	Forward	CAACGGCAAGACCAAAGGA	52.63	62.16
			Reverse	CCTGGAAGAGGCGGGTATG	63.16	62.86
RsDOT1	XM_018625501.1	243	Forward	CTGGCATCGGCATAGGTCTC	60.00	63.03
			Reverse	CACCACCACCTCCTCTTCCA	60.00	63.48
RsRDL5	XM_018609107.1	189	Forward	CGTTGGTTATCGGCTTGGTT	50.00	62.13
			Reverse	GTCACTGCTCCTTCCTTCCTC	57.14	60.39
**Genes of *CLE41*-associated pathway**						
RsWOX4-1	XM_018631720.1	177	Forward	ACAAGGTGGAACCCGACTCA	55.0	54.1
			Reverse	TCTCTCCCGGGCTTTGTGGT	60	58.1
RsWOX14	XM_018626710.1	202	Forward	CTCCTCCACAACTCTCTCTCTTC	52.2	50.6
			Reverse	ATCCTACGTCGGTTCGGTGTTCC	56.5	60.0
RsPXY	XM_018627910.1	232	Forward	TCCTTCTTCTTCCTTTTTCTTCCTT	36.00	61.28
			Reverse	GGGAGGTTGCCTGTGAGGTT	60.00	63.70
**Reference genes**						
RsGAPDH	XM_018627555.1	199	Forward	TCTCTTCGGTGAGAAGCCAGTCA	51.9	57.8
			Reverse	TCAAGTGAGCAGCAGCCTTGTC	51.6	57.0
RsUBQ	XM_018620331.1	198	Forward	ACTTGGTCCTCAGGCTTCGTGGT	53.7	59.9
			Reverse	AAAGATCAACCTCTGCTGGTCCG	51.9	58.3
